# AIAP: A Quality Control and Integrative Analysis Package to Improve ATAC-seq Data Analysis

**DOI:** 10.1016/j.gpb.2020.06.025

**Published:** 2021-07-15

**Authors:** Shaopeng Liu, Daofeng Li, Cheng Lyu, Paul M. Gontarz, Benpeng Miao, Pamela A.F. Madden, Ting Wang, Bo Zhang

**Affiliations:** 1 Department of Developmental Biology, Center of Regenerative Medicine, Washington University School of Medicine, St. Louis, MO 63108, USA; 2 Department of Genetics, Center for Genomic Sciences and Systems Biology, Washington University School of Medicine, St. Louis, MO 63108, USA; 3 Department of Psychiatry, Washington University School of Medicine, St. Louis, MO 63108, USA

**Keywords:** ATAC-seq, Quality control, Chromatin accessibility, Differential analysis, Data visualization

## Abstract

Assay for transposase-accessible chromatin with high-throughput sequencing (**ATAC-seq**) is a technique widely used to investigate genome-wide **chromatin accessibility**. The recently published Omni-ATAC-seq protocol substantially improves the signal/noise ratio and reduces the input cell number. High-quality data are critical to ensure accurate analysis. Several tools have been developed for assessing sequencing quality and insertion size distribution for ATAC-seq data; however, key **quality control** (QC) metrics have not yet been established to accurately determine the quality of ATAC-seq data. Here, we optimized the analysis strategy for ATAC-seq and defined a series of QC metrics for ATAC-seq data, including reads under peak ratio (RUPr), background (BG), promoter enrichment (ProEn), subsampling enrichment (SubEn), and other measurements. We incorporated these QC tests into our recently developed ATAC-seq Integrative Analysis Package (AIAP) to provide a complete ATAC-seq analysis system, including quality assurance, improved peak calling, and downstream **differential analysis**. We demonstrated a significant improvement of sensitivity (20%–60%) in both peak calling and differential analysis by processing paired-end ATAC-seq datasets using AIAP. AIAP is compiled into Docker/Singularity, and it can be executed by one command line to generate a comprehensive QC report. We used ENCODE ATAC-seq data to benchmark and generate QC recommendations, and developed *qATACViewer* for the user-friendly interaction with the QC report. The software, source code, and documentation of AIAP are freely available at https://github.com/Zhang-lab/ATAC-seq_QC_analysis.

## Introduction

To regulate the transcription of a eukaryotic genome, chromatin must remain in an accessible state to allow binding of transcription factors and initiation of transcription activation [1], [2], [3], [4]. Several sequencing-based methods have been developed to assess chromatin accessibility and nucleosome positioning, including DNase I hypersensitive sites sequencing (DNase-seq) [5], formaldehyde-assisted isolation of regulatory elements with sequencing (FAIRE-seq) [6], micrococcal nuclease digestion with deep sequencing (MNase-seq) [7], and the recently developed Assay for Transposase-accessible Chromatin with high-throughput sequencing (ATAC-seq) [8]. ATAC-seq can detect the accessible regions of a genome by identifying open chromatin regions (OCRs) using a prokaryotic Tn5 transposase [8], [9], and the technology features an easy experimental protocol, a reduced requirement of input material, and a high signal/noise ratio. These unique advantages have propelled ATAC-seq technology to quickly become a widely-used method to define chromatin accessibility, especially in several large consortiums focusing on functional genomics profiling, including ENCODE [10], TaRGET II [11], and IHEC [12].

The ATAC-seq analysis strategy is primarily adopted from ChIP-seq data analysis. After aligning sequencing reads to the genome, peak calling tools, such as MACS2 [13], are commonly used to identify highly enriched ATAC-seq signals across the genome. Unlike ChIP-seq, an ATAC-seq experiment does not normally require input control. Thus, accurately assessing the quality of ATAC-seq data is a critical step influencing downstream analysis. Several software packages were developed for ATAC-seq quality control (QC) and data analysis [14], [15], [16]. These tools provide general QC metrics of sequencing data, including read quality score, sequencing depth, duplication rate, and library insert fragment size distribution. Many tools also provide analysis functions, including footprinting analysis, motif analysis, and library complexity analysis.

Here, we present ATAC-seq Integrative Analysis Package (AIAP), a software package containing an optimized ATAC-seq data QC and analysis pipeline. Along with general QC metrics, such as library insert fragment size distribution, we specifically introduced a series of QC metrics for ATAC-seq, including reads under peak ratio (RUPr), background (BG), promoter enrichment (ProEn), subsampling enrichment (SubEn), and other measurements. By applying AIAP, we demonstrated a significant improvement in both peak calling and differential analysis by processing the paired-end sequencing data in single-end mode: more than 20% of ATAC-seq peaks can be identified using AIAP, and over 30% more differentially accessible regions (DARs) can be identified by AIAP in downstream analysis. We applied AIAP to reanalyze 70 mouse ENCODE [17] ATAC-seq datasets and determined the general QC recommendations for ATAC-seq data analysis. We also developed *qATACViewer*, a visualization tool included in AIAP, for user-friendly visualization of QC reports. AIAP is compiled into a Docker/Singularity image to allow maximized compatibility on different operating systems and computing platforms. The software, source code, and documentation are freely available at https://github.com/Zhang-lab/ATAC-seq_QC_analysis.

## Method

**Figure 1:**
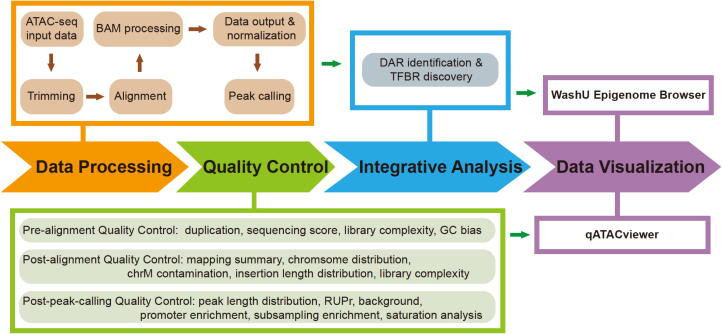
**Schematic representation of AIAP** The schema reports the four analytical steps, namely, Data Processing, Quality Control, Integrative Analysis, and Data Visualization. AIAP, ATAC-seq Integrative Analysis Package; RUPr, reads under peak ratio; DAR, differentially accessible region; TFBR, transcription factor binding region.

Here, we describe AIAP for processing and analyzing ATAC-seq data. The AIAP workflow typically consists of four steps, as shown in Figure 1: 1) Data Processing; 2) QC; 3) Integrative Analysis; and 4) Data Visualization. Below, we introduce the technical details of AIAP. Detailed documentation is available at https://github.com/Zhang-lab/ATAC-seq_QC_analysis/.

## ATAC-seq data processing

The data processing step first configures the working path. The ATAC-seq paired-end (PE) raw-read FASTQ files are trimmed by *Cutadapt* and aligned to the reference genome by *BWA* [18]. The BAM file is further processed by *methylQA* [19] in the ATAC mode. The *methylQA* first filters unmapped and low-quality mapped PE reads and then identifies the Tn5 insertion position at each read end by shifting + 4 bp/−5 bp on the positive/negative strands. *methylQA* further extends 75 bp in both directions around the Tn5 insertion position to create two pseudo single-end (SE) mapped reads with the length of 150 bp in PE as SE (PE-asSE) mode. Next, AIAP compiles different files for downstream analysis (.bed files) and normalized visualization (.bigWig files). The bed file is used to perform peak calling by MACS2 [13] with a q value cut-off of 0.01 and the following setting: *--keep-dup 1000 --nomodel --shift 0 --extsize 150*.

In PE-noShift mode, AIAP first filters unmapped and low-quality mapped PE reads and then identifies the Tn5 insertion position at each read end by shifting + 4 bp/−5 bp on the positive/negative strands, then isolates the whole fragment between two insertion positions to create one PE mapped fragment for downstream analysis (.bed files) and normalized visualization (.bigWig files). The bed file is used to perform peak calling by MACS2 [13] with a q value cut-off of 0.01 and the following setting: *--keep-dup 1000 --nomodel --shift 0 --extsize 0*.

## ATAC-seq data QC

AIAP performs a series of quality checking steps before and after alignment. AIAP calls *FastQC* to check the sequencing quality, duplication rate, and GC bias before alignment. After alignment, AIAP generates the mapping statistics summary, chromosome distribution of uniquely mapped reads, mitochondrial genome (chrM) contamination rate, library insert fragment size distribution, and library complexity. AIAP also performs a series of post-peak calling quality checks, including peak width distribution, RUPr, BG, ProEN, and SubEn. AIAP also provides saturation analysis, promoter peak distribution, and signal ranking analysis. AIAP reports the quality metrics in a JSON file, which can be visualized using *qATACViewer*. The default QC standard of AIAP is determined by the QC metrics of mouse ATAC-seq data generated by ENCODE consortium (Good standard: Mean; Acceptable standard: Mean − SD). We defined the key QC metrics below.

## Alignment QC

There are 5 QC metrics for alignment. 1) Non-redundant uniquely mapped reads refer to the reads that are uniquely mapped to the reference genome after removing redundancy. 2) The second is chromosome distribution/chrM contamination rate. The former describes the distribution of uniquely mapped reads across all chromosomes, while for the latter, the number of uniquely mapped reads on chrM is used as a QC metric to measure the quality of the ATAC-seq library. 3) Library insert fragment size distribution is measured as the length of DNA fragment defined by non-redundant uniquely mapped read-pairs. 4) Library complexity is estimated in both duplication rate and predicted yield of distinct reads generated by *preseq* (https://github.com/smithlabcode/preseq). 5) To obtain the total number of useful single ends, each end of a non-redundant uniquely mapped read pair will be shifted + 4 bp/−5 bp on the positive/negative strands and then further extended 75 bp in both directions around the Tn5 insertion position.

## Peak calling QC

### Rupr

RUPr is defined as the percentage of all useful ends ($E_{\mathrm{total}}$) that fall into the called peak regions with at least 50% overlap ($E_{under\_peaks}$). RUPr is calculated as follows.

(1) $$\mathrm{RUPr}=\frac{E_{under\_peaks}}{E_{\mathrm{total}}}$$

## Background

In total, 50,000 genomic regions (500 bp each) are randomly selected from the genome outside of ATAC-seq peaks. The ATAC-seq signal in each region is calculated as reads per kilobase per million mapped reads (RPKM). The percentage of all such regions with the ATAC-seq signal over the theoretical threshold (RPKM = 0.377) is considered high-background and used as a QC metric to indicate the background noise.

(2) $$\mathrm{Background}=\frac{number\_of\_regions\_with\_high\_background}{50,000}$$

## ProEN

The promoters, the regions +/− 1 kb around transcription start sites (TSSs) of active genes, provide a positive control for OCRs. The ATAC-seq useful ends enriched on detected promoters (ATAC-seq peaks) are used as a QC metric to measure the signal enrichment calculated as follows.

(3) $$\mathrm{ProEn}=\frac{E_{under\_promoter\_peaks}/Length_{total\_promoter\_peaks}}{E_{\mathrm{total}}/Length_{\mathrm{genome}}}$$

## SubEn

The ATAC-seq signal (useful ends) enriched on the detected ATAC-seq peaks is used as a QC metric to measure the signal enrichment at the genome-wide level. To avoid sequencing-depth bias, 10 million useful ends are sampled from the complete dataset, and peak calling is performed to identify the OCRs. SubEn is calculated after 10 million pseudo counts are added into the calculation as background, which can avoid calculation failure caused by the low sequencing depth of testing ATAC-seq library.

(4) $$\mathrm{SubEn}=\frac{\frac{E_{under\_peaks}}{\mathrm{Lengt}h_{total\_peaks}}+\frac{10million}{\mathrm{Lengt}h_{\mathrm{genome}}}}{\left(E_{under\_peaks}+10million\right)/\left(Length_{\mathrm{genome}}-Length_{total\_peaks}\right)}$$

## Saturation analysis

MACS2 is used to call narrow peaks for a series of subsampling from complete useful ends with a step of 10% of total sequencing depth. The length of identified peaks covering genomic regions at each subsampling are used to calculate the recovery (percentage) of complete peaks covering genomic regions when using complete useful ends.

## Signal ranking analysis

The ATAC-seq peak signals are ranked, and the percentage of promoter peaks in each quantile is determined.

## ATAC-seq data integrative analysis

AIAP includes two downstream analysis components: (1) analysis of DARs between two groups of samples and (2) discovery of transcription factor binding regions (TFBRs). AIAP calculates the read counts for all peaks identified under all conditions after peak calling, and a pair-wise comparison is performed by querying the *R* package *DESeq2* [20] based on the design table. AIAP will further identify potential TFBRs under ATAC-seq peaks by implementing the Wellington algorithm [21].

## ATAC-seq data and QC report visualization

AIAP generates a collection of files for visualizing the ATAC-seq data on a genome browser [22], [23], [24], including the normalized signal density file (normalized to 10 million total reads) in bigwig format, the Tn5 insertion position file in bigwig format, the peak file in bed format, and the footprint position file in bed format. AIAP generates a JSON QC report that can be visualized with the embedded *qATACViewer* (Figure S1).

## Calculation of DNase I hypersensitive sites and histone modification signal

The raw data FASTQ files for ATAC-seq and histone ChIP-seq were downloaded from ENCODE data portal (https://www.encodeproject.org/), and listed in Table S1. The ATAC-seq FASTQ files were processed by AIAP as described above. The ChIP-seq FASTQ files were aligned to the mouse genome (mm10 assembly) and were further processed by *methylQA*. Methylation calling of whole-genome bisulfite sequencing (WGBS) data was downloaded from the ENCODE data portal. The averaged signals of ATAC-seq, ChIP-seq, and WGBS were calculated at 100-bp windows within 5 kb around the center of the ATAC-seq peaks and were plotted in *R*. The processed DNase I hypersensitive site (DHS) data were downloaded from the ENCODE data portal.

## DAR identification

The DARs of each tissue were identified between different mouse developmental stages, embryonic day 11.5 (E11.5) and postnatal day 0 (P0), to evaluate the performance of AIAP. The ATAC-seq peaks generated by AIAP were used as test regions, the read counts were calculated in both PE-asSE and PE-noShift modes, and the DARs were identified as described above with adjusted *P* < 0.01 and absolute log_2_ FC > 1. In PE-asSE mode, two Tn5 insertion events of one read-pair were considered independent of each other, and one read-pair was divided into two SE fragments to represent two Tn5 insertion events. In PE-noShift modes, one read-pair was used as one single fragment for downstream analysis.

## Results

### Defining the QC metrics of ATAC-seq data

**Figure 2:**
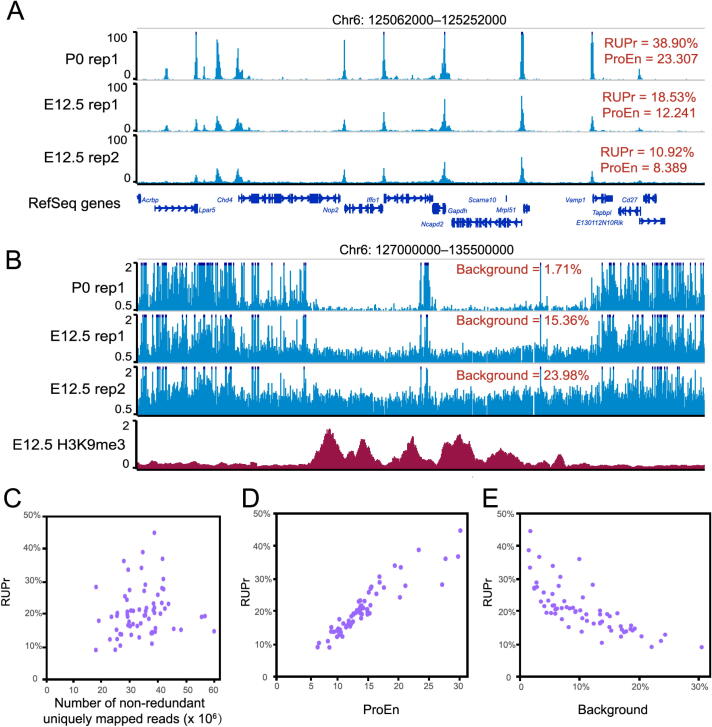
**Key QC metrics of ATAC-seq data A**. Forebrain ATAC-seq datasets (sequencing depth normalized to 10 million reads) with distinct RUPr and ProEn (top to bottom: high to low) were visualized on the WashU Epigenome Browser. **B**. Forebrain ATAC-seq datasets (same as A) with distinct background values (top to bottom: low to high) in the heterochromatin regions highly enriched with repressive H3K9me3 histone modification in the mouse forebrain. **C.** Relationship between the number of non-redundant uniquely mapped reads and RUPr in ENOCDE mouse ATAC-seq datasets. **D.** Relationship between ProEn and RUPr in ENOCDE mouse ATAC-seq datasets. **E**. Relationship between background and RUPr in ENOCDE mouse ATAC-seq datasets. QC, quality control; RUPr, reads under peak ratio; ProEn, promoter enrichment; E12.5, embryonic day 12.5; P0, postnatal day 0.

Conducting QC checks at different steps of data processing and correctly interpreting QC metrics are crucial to ensure a successful and meaningful analysis. Different QC metrics report important information regarding different aspects of genomic data; thus, it is essential to define the key QC metrics for ATAC-seq data before performing an analysis. In addition to the traditional QC metrics shown in Figure 1, we specifically chose RUPr, BG, and ProEn as key QC metrics to measure the quality of ATAC-seq data. RUPr is an essential QC metric for ChIP-seq experiments [25] and is widely adopted to measure ATAC-seq data. The ENCODE consortium recommends that at least 20% of non-redundant uniquely mapped reads be located in peak regions. A higher RUPr usually indicates a high signal-to-noise ratio. Similar to RUPr, a higher ProEn also indicates a high signal-to-noise ratio. ProEn is calculated to indicate the enrichment of the ATAC-seq signal over gene promoters, which are usually in open chromatin across different tissue and cell types. We used the ENCODE ATAC-seq data as a benchmark, and we determined that RUPr and ProEn directly reflect the quality of ATAC-seq at comparable sequencing depths (Figure 2**A**). The ATAC-seq peaks are sharper and stronger when the ATAC-seq data have high RUPr and ProEn values, suggesting better signal enrichment and better quality in the ATAC-seq experiments.

We further defined BG to directly measure the background noise level in the ATAC-seq experiments. We randomly selected 50,000 genomic regions (size: 500 bp) from regions of the genome that do not overlap with ATAC-seq peaks after peak calling. The ATAC-seq signals over each region are calculated as RPKM, and the regions with an ATAC-seq signal over a theoretical threshold (RPKM = 0.377) are considered high-background regions. The percentage of high-background regions within 50,000 randomly selected genomic regions is used as a QC metric to measure the background noise of the ATAC-seq data. We noticed that different background noise levels were directly reflected by the QC metric of background, especially in the heterochromatin regions, which are enriched with H3K9me3 signals (Figure 2B).

To further explore the QC metrics, we used AIAP to process 70 ATAC-seq datasets generated by the ENCODE consortium (Table S2). We specifically checked the key metrics, including RUPr, ProEn, SubEn, and BG. We noticed that these metrics were not dependent on sequencing depth (Figure 2C, Figure S2). RUPr was positively correlated with ProEn, proving an accurate measurement of signal enrichment in the ATAC-seq data (Figure 2D). BG was negatively correlated with RUPr, providing a measurement of the noise level in the ATAC-seq data (Figure 2E).

## AIAP improves the sensitivity of discovering ATAC-seq peaks

**Figure 3:**
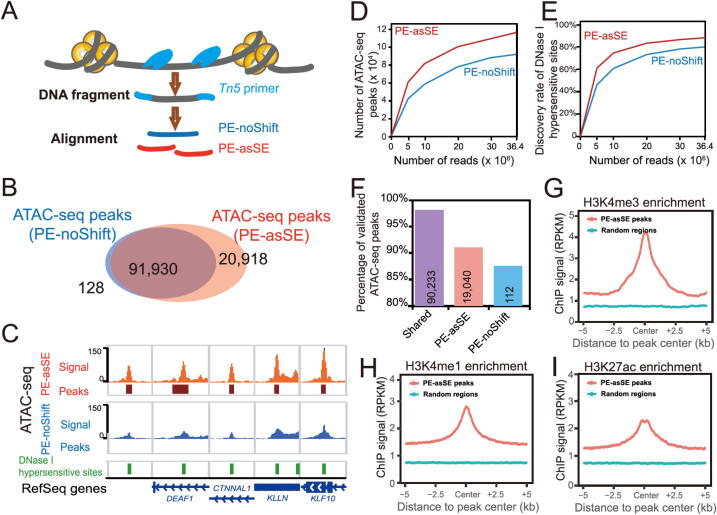
**AIAP data processing strategy (PE-asSE) performs better than classic method (PE-noShift) in OCR identification A.** Schematic representation of PE-noShift and PE-asSE data processing strategy. PE-noShift representing the one complete DNA fragment insertion was generated as in previous studies [26], [27], [28], [29], [30], [31], [32]. PE-asSE representing two DNA fragment insertions was generated by AIAP. **B.** Venn diagram showing ATAC-seq peaks identified with PE-noShift and PE-asSE in the GM12878 cell line. **C.** ATAC-seq data of GM12878 cell line were independently processed as PE-asSE (red) and PE-noShift (blue), and visualized together with DHSs of GM12878 cell line (green) on the WashU Epigenome Browser. Five regions were randomly selected that contain the peaks identified only in PE-asSE mode but not in PE-noShift mode. **D.** Number of ATAC-seq peaks identified with PE-noShift mode and PE-asSE mode at different sequencing depths by randomly sampling the Omni-ATAC-seq data of GM12878 cell line [31]. **E.** Discovery rate of DHSs of GM12878 cell line by PE-asSE and PE-noShift at different sequencing depths (randomly sampling same as in D). **F.** Percentage of shared, PE-asSE-specific, and PE-noShift-specific ATAC-seq peaks validated by DHSs of GM12878 cell line identified by ENCODE. PE-asSE-specific ATAC-seq peaks were highly enriched with active histone modifications H3K4me3 (**G**), H3K4me1 (**H**), and H3K27ac (**I**), when compared to 20,000 regions randomly selected from the genome (green). DHS, DNase I hypersensitive site.

To define ATAC-seq peaks, peak calling strategies adopted from ChIP-seq analysis are widely used to analyze ATAC-seq data. However, unlike ChIP-seq data, an ATAC-seq experiment does not have input control and is usually sequenced with the PE sequencing method to profile the size of DNA fragments. The uniquely aligned PE reads have been used to call open chromatin peaks after alignment in many studies [26], [27], [28], [29], [30], [31], [32]. After detecting the distribution of reads with different lengths under the peak regions, we noticed that the medium fragments and long fragments have similar distributions across the genome in ATAC-seq experiments (Table S3). Such evidence indicates that most of the captured fragment represents the open chromatin signal that can be derived from the Tn5 insertions in ATAC-seq experiments. To better represent the Tn5 insertion event, we shifted each end of the non-redundant uniquely mapped read pair + 4 bp/−5 bp on the positive/negative strands to define the Tn5 insertion position and then further extended 75 bp in both directions around the Tn5 insertion position. By applying this strategy, one non-redundant uniquely mapped read pair is divided into two SE fragments [PE-asSE], and the sequencing depth doubles compared to that of traditional analysis methods that manage PE fragments without shifting (PE-noShift) (Figure 3**A**).

To validate the sensitivity of our analysis strategy, we downloaded published ATAC-seq data of GM12878 cells generated by the Greenleaf laboratory with the Omni-ATAC-seq protocol [31]. We first processed the data by following the classical method based on non-redundant uniquely mapped PE reads (PE-noShift) and performed peak calling. In PE-noShift mode, we identified 92,058 narrow peaks. In parallel, we applied AIAP to process the same data in PE-asSE mode and performed peak calling with identical parameters (see Method), and 112,848 peaks were identified. Compared to PE-noShift mode, PE-asSE mode reported ∼ 99.9% of PE-noShift peaks and identified ∼ 23% additional peaks (20,918) (Figure 3B). By visually inspecting the signal density on the genome browser, we noticed that most of the PE-asSE-specific peaks overlapped with known ENCODE DHSs (Figure 3C). We examined the ATAC-seq peaks and known GM12878 DHSs obtained from the ENCODE data portal. We noticed that PE-asSE mode identified more ATAC-seq peaks at different sequencing depths (Figure 3D). When analyzing the full GM12878 Omni-ATAC-seq dataset, PE-noShift mode identified ∼ 80% of DHSs, and PE-asSE mode identified ∼ 85% of DHSs (Figure 3E). We further used merged DHSs of 95 cell lines to measure the specificity of identified ATAC-seq peaks. We found that nearly 98% of common peaks identified by both PE-asSE and PE-noShift modes overlapped with known DHSs. A total of 19,040 out of 20,918 peaks identified only by PE-asSE mode overlapped with known DHSs, and 112 out of 128 peaks identified only by PE-noShift mode overlapped with known DHSs (Figure 3F). We also used DHS data to estimate the Type-I and Type-II errors of AIAP. The ATAC-seq peaks that cannot be validated by known DHSs are considered as potential false positive. In total 3575 out of 112,848 ATAC-seq peaks in AIAP PE-asSE mode and 1713 out of 92,058 ATAC-seq peaks in AIAP PE-noShift mode are considered as potential Type-I errors (false discovery rate: 3.17% and 1.86%, respectively). However, considering the relatively lower sensitivity of DNase-seq when comparing to ATAC-seq assay, we believe the Type-I error calculated here was overestimated. We further defined the 39,205 GM12878 DHSs that were commonly identified in two independent replicates as true positive DHSs. AIAP PE-asSE can identify 38,078 of them, and 1127 DHSs were considered as negative (Type-II errors) with a false negative rate of 2.87%. Meanwhile, AIAP PE-noShift mode predicted 1827 DHSs as negative (Type-II errors) with a false negative rate of 4.66%. These results suggest that PE-asSE mode can greatly improve the sensitivity of the OCR discovery.

ATAC-seq peaks are generally considered regulatory elements that are enriched for specific histone modifications. We downloaded ChIP-seq data of GM12878 histone modifications (H3K4me3, H3K4me1, and H3K27ac) that are associated with promoter and enhancer activities to validate the functionality of PE-asSE-specific ATAC-seq peaks. Compared to randomly selected genomic regions, the PE-asSE-specific ATAC-seq peaks were highly enriched in all active histone modifications (Figure 3G–I). These results suggest that the PE-asSE-specific ATAC-seq peaks are functional regulatory elements rather than false positives. We further utilized AIAP to analyze the ATAC-seq data of multiple tissues. Compared to the classic PE-noShift mode, the PE-asSE mode resulted in a 28%–55% increase in sensitivity when processing the ATAC-seq data (Table S4). These results indicate that AIAP can dramatically enhance the sensitivity of OCR discovery with high specificity.

## AIAP improves the sensitivity of DAR identification

**Figure 4:**
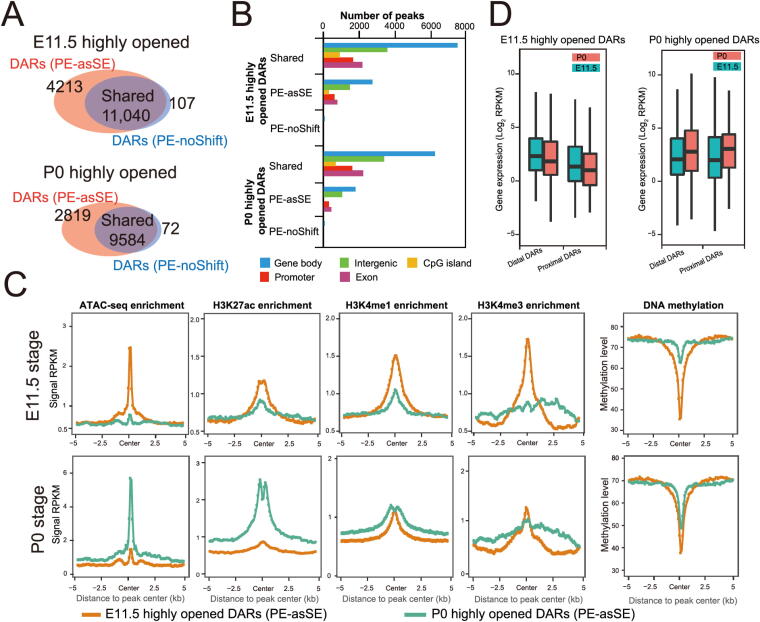
**AIAP data processing strategy (PE-asSE) performs better than the classic method (PE-noShift) in DAR identification A.** Venn diagram showing DARs identified with PE-noShift mode and PE-asSE mode when comparing ATAC-seq data collected from mouse liver at E11.5 and those from P0. **B.** Genomic distribution of 20,624 shared, 7032 PE-asSE-specific, and 179 PE-noShift-specific DARs. **C.** Enriched epigenetic modifications (left to right: ATAC-seq, H3K27ac ChIP-seq, H3K4me1 ChIP-seq, H3K4me3 ChIP-seq, and DNA methylation) on the PE-asSE-specific DARs from mouse liver at E11.5 (top) and P0 (bottom). **D**. The expression of genes associated with PE-asSE-specific E11.5 DARs (left) and P0 DARs (right) in proximal (2 kb around TSSs) and distal (2–20 kb around TSSs) at the two developmental stages. TSS, transcription start site.

Chromatin accessibility is dynamically associated with cellular responses to developmental cues, disease progression, and environmental stimuli. The identification of DARs has become an important approach to monitor the activity changes of regulatory elements [33]. Since AIAP dramatically increased the sensitivity of OCR discovery, we further tested the sensitivity of AIAP in identifying DARs. We downloaded ATAC-seq dataset of mouse liver E11.5 and P0 stages from the ENCODE data portal and processed these data in both PE-asSE and PE-noShift modes. As expected, PE-asSE mode identified 30% more ATAC-seq peaks than PE-noShift mode (Table S4). To test the sensitivity of DAR identification, we used the complete set of ATAC-seq peaks identified in PE-asSE mode and calculated the read counts based on both PE-asSE and PE-noShift modes (see Method). A total of 11,040 E11.5-specific and 9584 P0-specific DARs were identified by both modes (shared DARs). We also identified 4213 E11.5-specific and 2819 P0-specific DARs by using only PE-asSE mode (Figure 4**A**). Correspondingly, only 107 E11.5-specific and 72 P0-specific DARs were found by using only PE-noShift mode. Compared to PE-noShift mode, PE-asSE mode resulted in an ∼ 35% increase in the number of DARs identified. We further tested AIAP on other tissues at two developmental stages and found that AIAP identified 32%–168% more DARs in different tissues (Table S5).

We examined the genomic distribution of DARs and noticed that the distribution of PE-asSE-specific DARs had a similar distribution as shared DARs, that is, most DARs were located in intergenic and intronic regions, which is consistent with their potential enhancer functionality (Figure 4B). Because dynamic changes in chromatin accessibility accompany the alteration in epigenetic modification synchronously [1], [8], [28], [34], we further used epigenetic data from the same samples to validate the accuracy of the DARs. We first checked the epigenetic modifications around PE-asSE-specific DARs. Compared to P0-specific PE-asSE-specific DARs, the E11.5-specific DARs recruited highly active histone modifications associated with regulatory elements specifically at the E11.5 stage but not the P0 stage, including H3K27ac, H3K4me1, and H3K4me3 (Figure 4C, top). In contrast, the P0-specific PE-asSE-specific DARs recruited highly active histone modification H3K27ac specifically at the P0 stage but not H3K4me1 or H3K4me3 modification (Figure 4C, bottom). We also noticed that the E11.5-specific DARs were much less methylated at the E11.5 stage, but P0-specific DARs remained methylated at the E11.5 stage. However, E11.5-specific DARs were still un-methylated at the later postnatal stage. The observation of the loss of DNA methylation on regulatory elements during embryo development is consistent with that of a previous study [35]. The epigenetic modifications of shared DARs showed very similar patterns to the PE-asSE-specific DARs (Figure S3). We further examined the expression of genes around the identified DARs. The DARs were assigned to the nearest gene based on distance and were classified into a proximal group (2 kb around the TSS) and a distal group (2–20 kb around the TSS). We noticed that the expression of genes around PE-asSE embryonic DARs was downregulated during liver development. In contrast, the expression of genes around postnatal DARs was upregulated at the same time (Figure 4D).

## Discussion

AIAP is a new tool to perform quality assurance and downstream analysis of ATAC-seq data. Comparing with other tools (Table S6), AIAP provides a rapid and reliable data processing solution for ATAC-seq data. Using public datasets, we systematically tested the QC metrics of ATAC-seq data and established key QC metrics of ATAC-seq data. We determined that RUPr, ProEn, and BG were important measurements to estimate the quality of ATAC-seq data. All three QC metrics directly reflect the quality of library preparation, and the failure of these QC metrics reflect the low-quality of the ATAC-seq data, which cannot be corrected by merely increasing sequencing depth. We found that the RUPr and ProEn reflect the ATAC-seq signal enrichment, and BG indicates the overall background noise of the data. By combining these QC metrics, we obtained an accurate estimation of the quality of ATAC-seq data. We used AIAP to process 54 mouse ATAC-seq datasets to test and evaluate the QC metrics and generate the range of the QC metrics (Table S2). These ranges of QC metrics can be used as a reference to evaluate the success of ATAC-seq experiments.

We optimized the widely used classic analysis methodology and specifically used PE-asSE mode to process the PE sequenced ATAC-seq data. AIAP aligns the PE ATAC-seq data in PE mode to increase the alignment accuracy, and the BAM file is further processed in SE mode for downstream analysis. In PE-asSE mode, AIAP doubles the sequencing depth and dramatically increases the sensitivity of OCR identification. In our test, AIAP identified 20%–40% more ATAC-seq peaks than the widely used classic analysis methods. We further used corresponding DHS data and histone modification data to validate the specificity of newly identified ATAC-seq peaks by AIAP; most of the novel ATAC-seq peaks identified by AIAP were independently validated with DHSs and enriched for active histone modifications. Such a result indicates the high true positive rate resulting from the AIAP analysis strategy.

We also suggest that the PE-asSE strategy can improve the sensitivity of discovering DARs, which are wildly used to measure chromatin dynamics [36]. By using ENCODE ATAC-seq data of liver embryo development, we found that AIAP can identify 32%–168% more chromatin DARs than the classic PE-noShift mode. We further indicated that the novel DARs identified by AIAP were enriched in active histone modifications at different developmental stages. The E14.5-specific DARs were lowly methylated, and the H3K27ac signals were significantly enriched only in the E14.5 stage but not in the P0 stage. In contrast, the P0-specific DARs were highly methylated in the E14.5 stage without active histone modifications and became minimally methylated and recruited strong active H3K27ac signals in the P0 stage. We also noticed that the expression of genes around the developmental stage-specific DARs was associated with the openness of DARs, as other studies reported [37], [38]. These results suggest that AIAP can greatly improve the sensitivity to identify DARs with high specificity.

Finally, we compiled AIAP into a Docker/Singularity image to facilitate the easy operation of AIAP on high-performance computing clusters. AIAP can complete QC checking and file processing in ∼ 2 h for one typical ATAC-seq dataset, of 37 million PE reads (Table S7). Besides the high performance, AIAP also has a much better sensitivity and higher specificity (Figure 3F) when comparing to other tools, including ENCODE pipeline (Table S8). AIAP supports multiple genome assemblies, including human (hg19 and hg38), mouse (mm9 and mm10), and rat (rn6). Additionally, each step for QC and data processing is componentized and can be called by advanced users to build pipelines for specialized applications, and different genome assemblies can be easily and directly compiled for ATAC-seq data processing in other species.

Code availability.

The software, source code, and documentation of AIAP are freely available at https://github.com/Zhang-lab/ATAC-seq_QC_analysis.

Competing interests.

The authors have declared no competing interests.

### CRediT authorship contribution statement

**Shaopeng Liu:** Methodology, Software, Formal analysis, Resources, Writing – original draft. **Daofeng Li:** Methodology, Software, Visualization. **Cheng Lyu:** Methodology, Software, Formal analysis. **Paul M. Gontarz:** Formal analysis, Resources. **Benpeng Miao:** Investigation, Software, Formal analysis. **Pamela A.F. Madden:** Supervision, Project administration. **Ting Wang:** Supervision, Project administration, Writing – original draft, Writing – review & editing. **Bo Zhang:** Conceptualization, Methodology, Software, Formal analysis, Investigation, Supervision, Project administration, Writing – original draft, Writing – review & editing, Visualization.

## Supplementary material

Supplementary Figure S1 **ATAC-seq QC report visualized by qATACViewer for 3 selected ATAC-seq datasets A.** Summary of data and figure configuration. **B.** Mapping summary. **C.** Percentage of uniquely mapped reads on chrM, chrX, and chrY. **D.** Percentage of uniquely mapped reads on all the autosomes. **E.** Library complexity estimation by FastQC (before alignment) and PreSeq (after alignment). **F.** The length distribution of inserted DNA fragments. **G.** Promoter enrichment and subsampling enrichment. **H.** Background. **I.** The number of reads under the peak (left), and the number of peaks in gene’s promoter (red) and the number of peaks out of promoter (yellow). **J.** RUPr calculated as the percentage of reads under peaks. **K.** The length distribution of all peaks. **L.** Saturation analysis result.

Supplementary Figure S2 **Key QC metrics with subsampling test** Left: promoter enrichment; middle: reads under peak ratio; right: subsampling enrichment.

Supplementary Figure S3 **Enriched epigenetic modifications on the shared DARs between PE-noShift mode and PE-asSE mode** Left to right: ATAC-seq, H3K27ac ChIP-seq, H3K4me1 ChIP-seq, H3K4me3 ChIP-seq, DNA methylation. Top: embryonic day 11.5; Bottom: postnatal day 0. DAR, differentially accessible region.

Supplementary Table S1 **List of datasets used in this study**

Supplementary Table S2 **QC metrics of 70 mouse ENCODE ATAC-seq datasets**

Supplementary Table S3 **DNA insertion fragment distribution under the peak**

Supplementary Table S4 **Peak calling comparison between PE-asSE and PE-noShift**

Supplementary Table S5 **Differential analysis comparison between PE-asSE and PE-noShift**

Supplementary Table S6 **Performance comparison between AIAP and known ATAC-seq data processing pipelines**

Supplementary Table S7 **Benchmarking of AIAP and ENCODE ATAC-seq pipeline**

Supplementary Table S8 **Peak comparison between AIAP and ENCODE pipeline**
