# Supplementary material for: AIAP: A Quality Control and Integrative Analysis Package to Improve ATAC-seq Data Analysis
Source: Genomics Proteomics Bioinformatics. 2021 Jul 15;19(4):641–51. doi: 10.1016/j.gpb.2020.06.025 (PMC9040017; doi:10.1016/j.gpb.2020.06.025)
Supplement: Supplementary Figure S1 — ATAC-seq QC report visualized by qATACViewer for 3 selected ATAC-seq datasets A. Summary of data and figure configuration. B. Mapping summary. C. Percentage of uniquely mapped reads on chrM, chrX, and chrY. D. Percentage of uniquely mapped reads on all the autosomes. E. Library complexity estimation by FastQC (before alignment) and PreSeq (after alignment). F. The length distribution of inserted DNA fragments. G. Promoter enrichment and subsampling enrichment. H. Background. I. The number of reads under the peak (left), and the number of peaks in gene’s promoter (red) and the number of peaks out of promoter (yellow). J. RUPr calculated as the percentage of reads under peaks. K. The length distribution of all peaks. L. Saturation analysis result. [file mmc1.pdf]

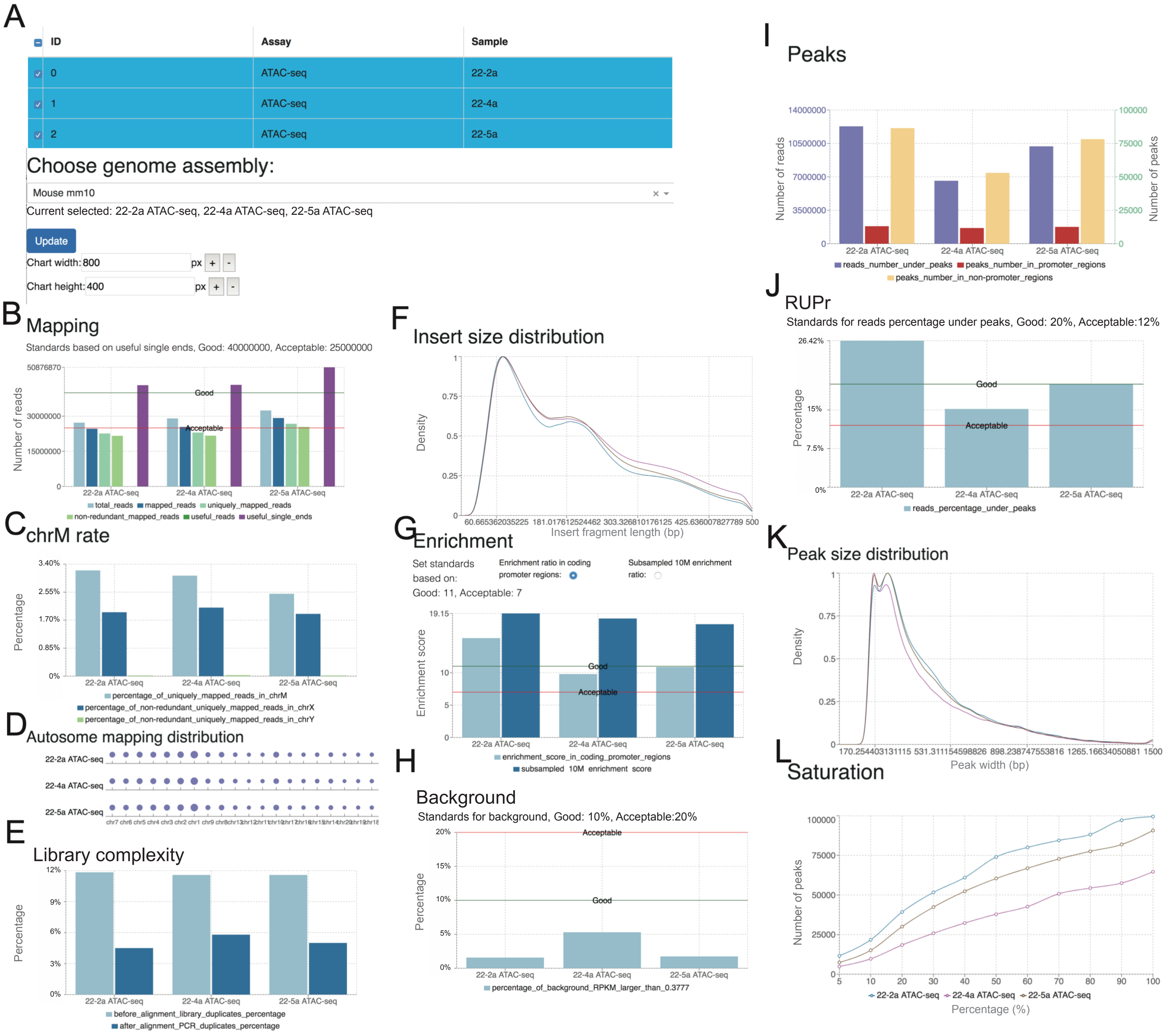
A
ID
Assay
Sample
0
ATAC-seq
22-2a
1
ATAC-seq
22-4a
2
ATAC-seq
22-5a
Choose genome assembly:
Mouse mm10
Current selected: 22-2a ATAC-seq, 22-4a ATAC-seq, 22-5a ATAC-seq
Update
Chart width: 800 px + -
Chart height: 400 px + -
B
Mapping
Standards based on useful single ends, Good: 40000000, Acceptable: 25000000
Number of reads
50876870
30000000
15000000
0
Good
Acceptable
22-2a ATAC-seq
22-4a ATAC-seq
22-5a ATAC-seq
total_reads
mapped_reads
uniquely_mapped_reads
non-redundant_mapped_reads
useful_reads
useful_single_ends
C
chrM rate
Percentage
3.40%
2.55%
1.70%
0.85%
0%
percentage_of_uniquely_mapped_reads_in_chrM
percentage_of_non-redundant_uniquely_mapped_reads_in_chrX
percentage_of_non-redundant_uniquely_mapped_reads_in_chrY
D
Autosome mapping distribution
22-2a ATAC-seq
22-4a ATAC-seq
22-5a ATAC-seq
E
Library complexity
Percentage
12%
9%
6%
3%
0%
before_alignment_library_duplicates_percentage
after_alignment_PCR_duplicates_percentage
F
Insert size distribution
Density
1
0.75
0.5
0.25
0
60.665362035225
181.017612524462
303.326810176125
425.636007827789
500
Insert fragment length (bp)
G
Enrichment
Set standards based on:
Good: 11, Acceptable: 7
Enrichment ratio in coding promoter regions:
Subsampled 10M enrichment ratio:
Enrichment score
19.15
10
5
0
Good
Acceptable
enrichment_score_in_coding_promoter_regions
subsampled_10M_enrichment_score
H
Background
Standards for background, Good: 10%, Acceptable:20%
Percentage
20%
15%
10%
5%
0%
Acceptable
Good
percentage_of_background_RPKM_larger_than_0.3777
I
Peaks
Number of reads
14000000
10500000
7000000
3500000
0
Number of peaks
100000
75000
50000
25000
0
reads_number_under_peaks
peaks_number_in_promoter_regions
peaks_number_in_non-promoter_regions
J
RUPr
Standards for reads percentage under peaks, Good: 20%, Acceptable:12%
Percentage
26.42%
15%
7.5%
0%
Good
Acceptable
reads_percentage_under_peaks
K
Peak size distribution
Density
1
0.75
0.5
0.25
0
170.254403131115
531.311154598826
898.238747553816
1265.16634050881
1500
Peak width (bp)
L
Saturation
Number of peaks
100000
75000
50000
25000
0
5
10
20
30
40
50
60
70
80
90
100
22-2a ATAC-seq
22-4a ATAC-seq
22-5a ATAC-seq
Percentage (%)
