# Supplementary material for: AIAP: A Quality Control and Integrative Analysis Package to Improve ATAC-seq Data Analysis
Source: Genomics Proteomics Bioinformatics. 2021 Jul 15;19(4):641–51. doi: 10.1016/j.gpb.2020.06.025 (PMC9040017; doi:10.1016/j.gpb.2020.06.025)
Supplement: Supplementary Figure S2 — Key QC metrics with subsampling test Left: promoter enrichment; middle: reads under peak ratio; right: subsampling enrichment. [file mmc2.pdf]

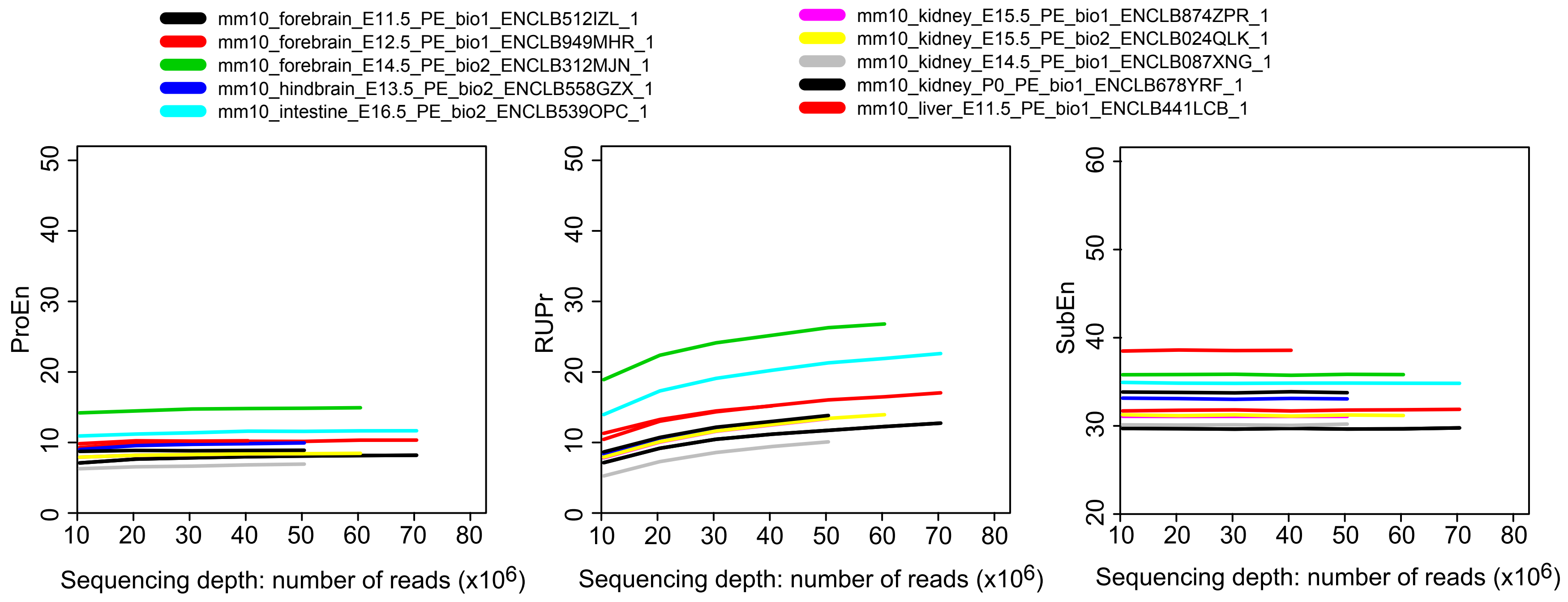
mm10_forebrain_E11.5_PE_bio1_ENCLB512IZL_1
mm10_forebrain_E12.5_PE_bio1_ENCLB949MHR_1
mm10_forebrain_E14.5_PE_bio2_ENCLB312MJN_1
mm10_hindbrain_E13.5_PE_bio2_ENCLB558GZX_1
mm10_intestine_E16.5_PE_bio2_ENCLB539OPC_1
mm10_kidney_E15.5_PE_bio1_ENCLB874ZPR_1
mm10_kidney_E15.5_PE_bio2_ENCLB024QLK_1
mm10_kidney_E14.5_PE_bio1_ENCLB087XNG_1
mm10_kidney_P0_PE_bio1_ENCLB678YRF_1
mm10_liver_E11.5_PE_bio1_ENCLB441LCB_1
ProEn
RUPr
SubEn
Sequencing depth: number of reads (x10^6)
Sequencing depth: number of reads (x10^6)
Sequencing depth: number of reads (x10^6)
