# Supplementary material for: AIAP: A Quality Control and Integrative Analysis Package to Improve ATAC-seq Data Analysis
Source: Genomics Proteomics Bioinformatics. 2021 Jul 15;19(4):641–51. doi: 10.1016/j.gpb.2020.06.025 (PMC9040017; doi:10.1016/j.gpb.2020.06.025)
Supplement: Supplementary Figure S3 — Enriched epigenetic modifications on the shared DARs between PE-noShift mode and PE-asSE mode Left to right: ATAC-seq, H3K27ac ChIP-seq, H3K4me1 ChIP-seq, H3K4me3 ChIP-seq, DNA methylation. Top: embryonic day 11.5; Bottom: postnatal day 0. DAR, differentially accessible region. [file mmc3.pdf]

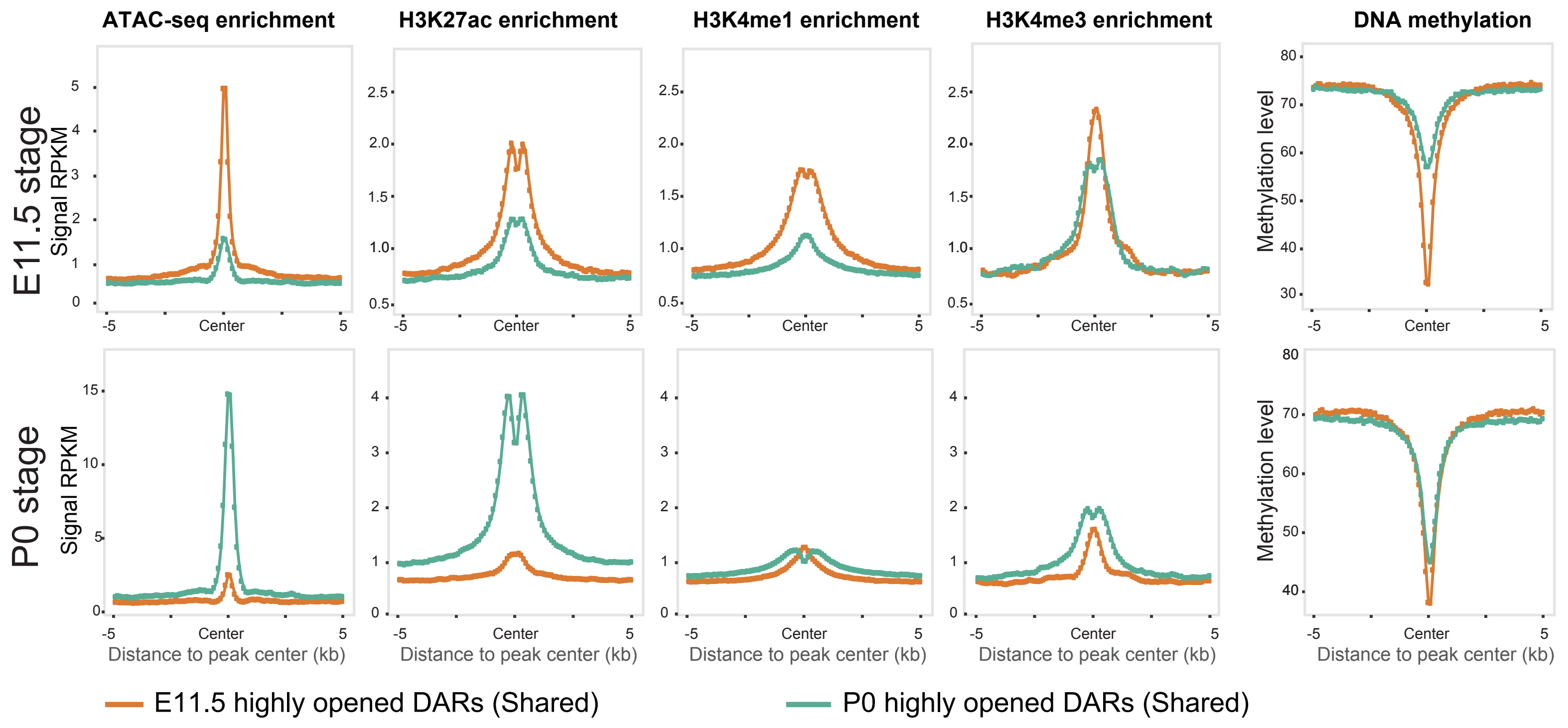
ATAC-seq enrichment
H3K27ac enrichment
H3K4me1 enrichment
H3K4me3 enrichment
DNA methylation
E11.5 stage
P0 stage
Signal RPKM
Methylation level
Center
-5
5
Distance to peak center (kb)
E11.5 highly opened DARs (Shared)
P0 highly opened DARs (Shared)
