# Supplementary material for: AIAP: A Quality Control and Integrative Analysis Package to Improve ATAC-seq Data Analysis
Source: Genomics Proteomics Bioinformatics. 2021 Jul 15;19(4):641–51. doi: 10.1016/j.gpb.2020.06.025 (PMC9040017; doi:10.1016/j.gpb.2020.06.025)
Supplement: Supplementary Table S3 — DNA insertion fragment distribution under the peak [file mmc6.docx]

**Table S3 DNA insertion fragment distribution under the peak**

|  | Forebrain | Intestine | Kidney | Liver | Lung | Stomach |
| --- | --- | --- | --- | --- | --- | --- |
| Sample ID | ENCLB042MOW | ENCLB362STB | ENCLB497HBT | ENCLB303HH | ENCLB080OEI | ENCLB490MG |
| Total No. of fragments | 24,914,597 | 22,141,337 | 26,597,998 | 16,120,650 | 33,646,778 | 17,254,895 |
| No. of short fragments(< 38 bp) | 25,334 | 36,920 | 106,029 | 21,373 | 55,781 | 31,901 |
| No. of medium fragments (38–150 bp) | 8,647,206 | 9,166,167 | 13,669,940 | 5,924,854 | 12,878,709 | 8,043,302 |
| No. of long fragments (> 150 bp) | 16,242,057 | 12,938,250 | 12,822,029 | 10,174,423 | 20,712,288 | 9,179,692 |
| RUP of medium fragments | 1,860,344 | 898,813 | 1,488,011 | 1,412,188 | 3,230,218 | 534,505 |
| RUP of long fragments | 2,804,650 | 1,235,315 | 1,174,004 | 2,060,910 | 5,062,100 | 456,521 |
| RUPr of medium fragments | 21.51% | 9.81% | 10.89% | 23.83% | 25.08% | 6.65% |
| RUPr of long fragments | 17.27% | 9.55% | 9.16% | 20.26% | 24.44% | 4.97% |

*Note*: RUP, reads under peak; RUPr: reads under peak ratio.
