# Supplementary material for: AIAP: A Quality Control and Integrative Analysis Package to Improve ATAC-seq Data Analysis
Source: Genomics Proteomics Bioinformatics. 2021 Jul 15;19(4):641–51. doi: 10.1016/j.gpb.2020.06.025 (PMC9040017; doi:10.1016/j.gpb.2020.06.025)
Supplement: Supplementary Table S4 — Peak calling comparison between PE-asSE and PE-noShift [file mmc7.docx]

**Table S4 Peak calling comparison between PE-asSE and PE-noShift**

|  | Forebrain | Intestine | | Kidney | | Liver | Lung | | | Stomach |  |
| --- | --- | --- | --- | --- | --- | --- | --- | --- | --- | --- | --- |
| Sample ID | ENCLB042MOW | ENCLB362STB | ENCLB497HBT | | ENCLB303HH | | | ENCLB080OEI | ENCLB490MG | | |
| No. of ATAC-seq peaks in PE-noShift | 39,127 | 22,644 | 25,437 | | 23,044 | | | 58,283 | 17,012 | | |
| No. of ATAC-seq peaks in PE-asSE | 49,993 | 33,548 | 39,259 | | 28,851 | | | 77,796 | 23,882 | | |
| No. of ATAC-seq peaks in PE-asSE validated by DNase-seq | 49,339 | 32,016 | 37,803 | | 27,795 | | | 75,517 | 23,083 | | |
| Percentage of validated ATAC-seq peaks | 98.69% | 95.43% | 96.29% | | 96.34% | | | 97.07% | 96.65% | | |
| No. of shared ATAC-seq peaks | 38,830 | 22,474 | 25,209 | | 22,924 | | | 57,987 | 16,881 | | |
| No. of PE(asSE)-specific ATAC-seq peaks | 11,163 | 11,074 | 14,050 | | 5927 | | | 19,809 | 7001 | | |
| Increase | 28.75% | 49.27% | 55.73% | | 25.85% | | | 34.16% | 41.47% | | |
