# Supplementary material for: AIAP: A Quality Control and Integrative Analysis Package to Improve ATAC-seq Data Analysis
Source: Genomics Proteomics Bioinformatics. 2021 Jul 15;19(4):641–51. doi: 10.1016/j.gpb.2020.06.025 (PMC9040017; doi:10.1016/j.gpb.2020.06.025)
Supplement: Supplementary Table S5 — Differential analysis comparison between PE-asSE and PE-noShift [file mmc8.docx]

**Table S5 Differential analysis comparison between PE-asSE and PE-noShift**

|  | Forebrain | Intestine | Kidney | Liver | Lung | Stomach |
| --- | --- | --- | --- | --- | --- | --- |
| Sample ID-1 | ENCLB252ZLC | ENCLB069VWJ | ENCLB087XNG | ENCLB200ODB | ENCLB517ADM | ENCLB880WNY |
| Sample ID-2 | ENCLB312MJN | ENCLB325XDP | ENCLB299QNB | ENCLB555EYH | ENCLB224FWZ | ENCLB322JPD |
| Sample ID-3 | ENCLB042MOW | ENCLB362STB | ENCLB678YRF | ENCLB303HHQ | ENCLB080OEI | ENCLB490MGZ |
| Sample ID-4 | ENCLB558DNK | ENCLB199HRA | ENCLB497HBT | ENCLB282VPH | ENCLB071NVJ | ENCLB105JML |
| Total No. of ATAC-seq peaks | 126,784 | 77,483 | 83,371 | 89,128 | 129,812 | 97,233 |
| No. of DARs in PE-noShift | 5113 | 7131 | 700 | 11,121 | 11,177 | 451 |
| No. of DARs in PE-asSE | 8464 | 10,808 | 1646 | 14,746 | 16,402 | 1211 |
| No. of shared DARs | 5084 | 7009 | 691 | 11,051 | 11,059 | 448 |
| No. of PE-asSE-specific DARs | 3351 | 3677 | 946 | 3625 | 5225 | 760 |
| Increase | 65.54% | 51.56% | 135.14% | 32.60% | 46.75% | 168.51% |

*Note*: DAR, differentially accessible region.
