# Supplementary material for: AIAP: A Quality Control and Integrative Analysis Package to Improve ATAC-seq Data Analysis
Source: Genomics Proteomics Bioinformatics. 2021 Jul 15;19(4):641–51. doi: 10.1016/j.gpb.2020.06.025 (PMC9040017; doi:10.1016/j.gpb.2020.06.025)
Supplement: Supplementary Table S6 — Performance comparison between AIAP and known ATAC-seq data processing pipelines [file mmc9.docx]

**Table S6 Comparison between *AIAP* and known ATAC-seq data processing pipelines**

|  | *AIAP* | ENCODE ATAC-seq pipeline | esATAC | ATACseqQC |
| --- | --- | --- | --- | --- |
| Distribute | Docker/Singularity | Cromwell/Docker/DNAnexus | R package | R package |
| Environment dependence | No | No | Yes | Yes |
| Preprocessing | FastQC | FastQC | FastQC | Not-included |
| Trimming | Cutadapt | Cutadapt | RemoveAdapter | Not-included |
| Alignment | BWA | Bowtie2 | Bowtie2 | Not-included |
| Post-alignment processing | methylQA | Samtools | Bedtools | Rsamtools |
| Peak calling | MACS2 | MACS2 | Fseq | MACS2 |
| QC output | JSON, qATACviewer | JSON, html | html | Text |
| BigWig output | Yes | Yes | Yes | No |
| Motif scanning | No | No | Yes | Yes |
| SNP analysis | No | No | No | Yes |
| GO enrichment | No | No | Yes | Yes |
| Differential analysis | Yes | No | Yes | Yes |
