# Supplementary material for: AIAP: A Quality Control and Integrative Analysis Package to Improve ATAC-seq Data Analysis
Source: Genomics Proteomics Bioinformatics. 2021 Jul 15;19(4):641–51. doi: 10.1016/j.gpb.2020.06.025 (PMC9040017; doi:10.1016/j.gpb.2020.06.025)
Supplement: Supplementary Table S7 — Benchmarking of AIAP and ENCODE ATAC-seq pipeline [file mmc10.docx]

**Table S7 Benchmarking of AIAP and ENCODE ATAC-seq pipeline**

|  | _AIAP (24 threads) | ENCODE(default, 1 thread) | ENCODE (24 threads) |
| --- | --- | --- | --- |
| Running time (h:min:s) | 2:08:23 | 20:08:23 | 6:33:55 |
| Memory usage | 11.48 GB | 0.86 GB | 0.88 GB |
| Size of output files | 9.4 GB | 38 GB | 38 GB |

*Note*: The dataset GM12878 Omni-ATAC-seq (SRA: SRP103230, total No. of reads: 37,596,063) is used for benchmarking, with computing environment setting: Intel Xeon CPU E5-2670 v3 @ 2.30GHz; 128GB RAM memory; and Ubuntu 16.04.5.
