# Supplementary material for: AIAP: A Quality Control and Integrative Analysis Package to Improve ATAC-seq Data Analysis
Source: Genomics Proteomics Bioinformatics. 2021 Jul 15;19(4):641–51. doi: 10.1016/j.gpb.2020.06.025 (PMC9040017; doi:10.1016/j.gpb.2020.06.025)
Supplement: Supplementary Table S8 — Peak comparison between AIAP and ENCODE pipeline [file mmc11.docx]

**Table S8 Peak comparison between AIAP and ENCODE pipeline**

|  | No. of peaks | Percentage |
| --- | --- | --- |
| AIAP peaks (*q* < 0.01) | 112,848 | --- |
| ENCODE peaks (*P* < 0.01, IDR) | 161,744 | --- |
| ENCODE peaks after removing redundancy (*P* < 0.01, IDR)^#^ | 67,646 | --- |
| ENCODE peaks (*P* < 0.01) identified by AIAP peaks (*q* < 0.01)* | 66,787 | 98.7% |
| AIAP peaks (*q* < 0.01) identified by ENCODE peaks (*P* < 0.01)* | 52,022 | 46.1% |

*Note*: ^#^: redundant lines in ENCODE IDR output peaks (*P* < 0.01) were removed by using “uniq” command. *: genomic region overlapping threshold > 50%. IDR, irreproducible discovery rate.
